# Correspondence: Reply to ‘Reassessing the contribution of natural gas to US CO_2_ emission reductions since 2007'

**DOI:** 10.1038/ncomms10693

**Published:** 2016-03-18

**Authors:** Kuishuang Feng, Steven J. Davis, Laixiang Sun, Klaus Hubacek

**Affiliations:** 1 Department of Geographical Sciences, University of Maryland, College Park, Maryland 20742, USA; 2 Department of Earth System Science, University of California, Irvine, California 92697, USA; 3 Department of Financial and Management Studies, SOAS, University of London, London WC1H 0XG, UK; 4 International Institute for Applied Systems Analysis (IIASA), Laxenburg A-2361, Austria

Our recent study^1^ quantified the drivers of US CO_2_ emissions between 1997 and 2013, with particular focus on the decline in emissions after 2007. Based on our findings, we argued that economic recession was more important than substitution of natural gas for coal in the power sector. In their comment, Kotchen and Mansur^2^ reevaluate and reinterpret our results to challenge this conclusion. Because their calculations, using two alternative methods, are consistent with our findings, here we respond to their alternative interpretation.

Kotchen and Mansur^2^ point out that by aggregating the influence of the six decomposed factors over the entire period 2007–2013, rather than 2-year intervals, the total contribution from changes in the fuel mix of the energy sector were greater than the contribution from changes in the volume of goods consumed. From this fact, they conclude that ‘the shale gas revolution has played a significant role' in the decrease of US emissions. We have three responses:

First, by aggregating over time, Kotchen and Mansur^2^ obscure the proximate cause of the decline in emissions, nearly all of which in fact occurred between 2007 and 2009, when changes in the volume of consumption were by far the dominant factor. But by equating a decrease in emissions with an avoided increase in emissions, Kotchen and Mansur^2^ neglect time-specific details to enhance the apparent contribution of shale gas: for example, since 2009, decreases in emissions due to changes in the fuel mix have not kept up with increases due to population growth, to say nothing of the economic recovery. During this period of recovery, changes in energy intensity, consumption patterns and production structure have been critical to keeping emissions down. These changes may also justify our emphasis of the recession as the key driver: a recent study showed that the historical response of emissions to economic contraction is asymmetrical, with long-term decreases in energy intensity and consumer behavior often characterizing successive periods of economic recovery^3^. While we might not witness another Great Recession in the near future, nor is it seen as a policy goal to reduce economic growth, we cannot depend on economic downturns to solve the CO_2_ emission problem and should mimic some of the structural changes following a recession.

Second, Kotchen and Mansur^2^ show that rather than fuel mix the change of production structure was the largest contributor (40%) over the entire period (2007–2013). Our result shows that this production structure change may be also due to the recession^1^. For example, the shares of inputs from energy-intensive industrial sectors such as chemicals, metal production and electricity declined substantially by 27%, 23% and 14%, respectively, during the recession (see Fig. 3 in ref. 1). Moreover, during the recession, companies had decreased willingness to invest in capital formation, leading to the share of inputs from chemicals, machinery and equipment, and construction sectors, which are all energy intensive, declining by 27%, 20% and 14%, respectively, in the total input.

Last, Kotchen and Mansur^2^ make the assumption that changes in the fuel mix are entirely the result of natural gas replacing coal for electricity generation. This is not the case. In fact, as we show in Fig. 1, the growth of electricity generated from low-carbon, renewable sources accounts for nearly half (47%) of the changes in the CO_2_ emissions intensity of US power sector between 2007 and 2013. This suggests that increased use of natural gas in the US contributed roughly 15% of the total observed decrease in US emissions. Further, between 2007 and 2009, when changes in the fuel mix contributed the most to decreased emissions (−2.3%, see Fig. 3 in ref. 1), renewables accounted for 65% of the change in power sector emissions intensity. While the relative role of natural gas has increased in recent periods (see Fig. 1), more renewable generating capacity might have been built if natural gas were less competitive^4^.

We stand by our conclusion that increased use of natural gas has contributed to the reduction of US emissions since 2007, but it is hardly the main driver of the decline.

## Additional information

**How to cite this article:** Feng, K. *et al*. Correspondence: Reply to ‘Reassessing the contribution of natural gas to US CO_2_ emission reductions since 2007'. *Nat. Commun*. 7:10693 doi: 10.1038/ncomms10693 (2016).

## Figures and Tables

**Figure 1:**
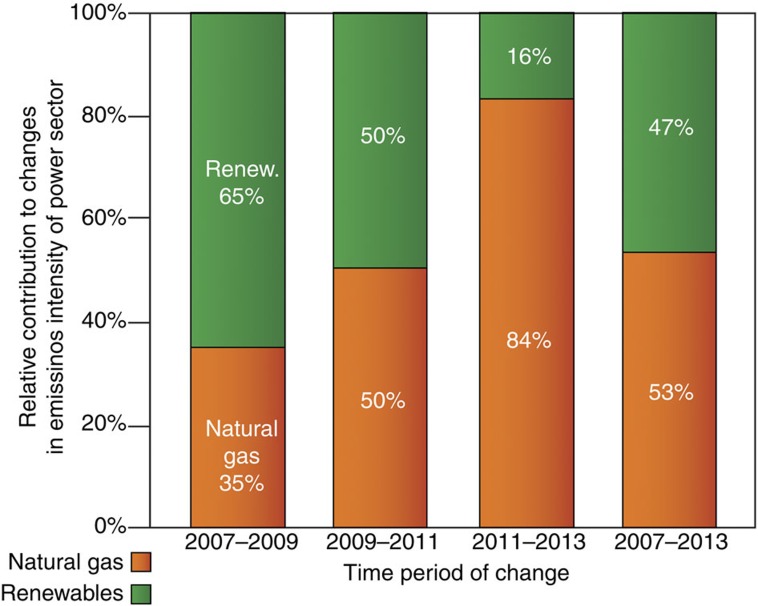
Relative contributions of natural gas and renewables to the changes in emissions intensity of US power sector.
